# Mitochondrial genomes of the freshwater monogonont rotifer *Brachionus fernandoi* and of two additional *B. calyciflorus* sensu stricto lineages from Germany and the USA (Rotifera, Brachionidae)

**DOI:** 10.1080/23802359.2022.2060765

**Published:** 2022-04-14

**Authors:** K. Kiemel, B. De Cahsan, S. Paraskevopoulou, G. Weithoff, R. Tiedemann

**Affiliations:** a Unit of Evolutionary Biology/Systematic Zoology, Institute for Biochemistry and Biology, University of Potsdam, Potsdam, Germany; b GLOBE Institute, University of Copenhagen, Copenhagen, Denmark; c School of Zoology, George S. Wise Faculty of Life Sciences, Tel Aviv University, Tel Aviv, Israel; d Unit of Ecology and Ecosystem Modelling, Institute for Biochemistry and Biology, University of Potsdam, Potsdam, Germany

**Keywords:** Mitogenome, cryptic species, *Brachionus calyciflorus* s.s., *Brachionus fernandoi*, monogonont rotifer

## Abstract

The *Brachionus calyciflorus* species complex was recently subdivided into four species, but genetic resources to resolve phylogenetic relationships within this complex are still lacking. We provide two complete mitochondrial (mt) genomes from *B. calyciflorus* sensu stricto (Germany, USA) and the mt coding sequences (cds) from a German *B. fernandoi*. Phylogenetic analysis placed our *B. calyciflorus* sensu stricto strains close to the published genomes of *B. calyciflorus*, forming the putative sister species to *B. fernandoi*. Global representatives of *B. calyciflorus* sensu stricto (i.e. Europe, USA, and China) are genetically closer related to each other than to *B. fernandoi* (average pairwise nucleotide diversity 0.079 intraspecific vs. 0.254 interspecific).

Recently, the freshwater rotifer *Brachionus calyciflorus* Pallas, 1766 species complex was – based on genetics and morphometrics – classified into four species: *Brachionus calyciflorus* sensu stricto Pallas, 1766, *Brachionus dorcas* Gosse, 1851, *Brachionus fernandoi* Michaloudi, Papakostas, Stamou, Neděla, Tihlaříková, Zhang & Declerck, 2018 and *Brachionus elevatus* Michaloudi, Papakostas, Stamou, Neděla, Tihlaříková, Zhang & Declerck, 2018 (Papakostas et al. 2016; Michaloudi et al. 2018) which show ecological differences, such as temperature adaptation (Paraskevopoulou et al. 2018). Several mitochondrial (mt) genomes of *Brachionus* rotifers are available: *B. plicatilis* Muller, 1786 (Suga et al. 2008), *B. koreanus* Hwang, Dahms, Park & Lee, 2013 (Hwang et al. 2014), *B. rotundiformis* Tschugunoff, 1921 (Kim et al. 2017), *B. rubens* Ehrenberg, 1838 (Choi et al. 2020), *B. paranguensis* Guerrero-Jiménez, Vannucchi, Silva-Briano, Adabache-Ortiz, Rico-Martínez, Roberts, Neilson & Elías-Gutiérrez, 2019 (Choi et al. 2019), and *B. angularis* Gosse, 1851 (Kim et al. 2020). For the *B. calyciflorus* species complex, two mtgenomes were published so far (Nie et al. 2016; Choi et al. 2019), but without taking into account the new species delimitations.

For the newly described species of the *B. calyciflorus* species complex, we provide two new mtgenomes of *B. calyciflorus* sensu stricto from hitherto untyped regions (IGB, Germany, exact origin unknown; Oneida Lake, USA, 43°12′49.4″N 75°55′31.7″W) and coding sequences (cds) of *B. fernandoi* (A10, Germany, 52°30′27.2″N 13°17′14.8″E). Samples were taken from public waters and do not require a permission. Samples are permanently stored at the University of Potsdam, Germany (https://www.uni-potsdam.de, Ralph Tiedemann, tiedeman@uni-potsdam.de) under the voucher numbers: IGB, Oneida and A10. Strains were cultured under laboratory conditions (20 °C in a 16:8 light:dark photoperiod, details in Paraskevopoulou et al. 2018) for more than 10 years. Individuals were filtered through a 30 µm sieve, re-suspended in WC medium (details in Paraskevopoulou et al. 2018) in a 50 mL tube and centrifuged at 2000 × *g* for 10 min to pellet phytoplankton and other debris, before transferring the rotifers into 300 µL of TRIzol LS and storing them at –80 °C. RNA was extracted using a customized Trizol/chloroform protocol and built into an Illumina NextSeq/HiSeq library using a NEXTflex Rapid Directional RNA-Seq Library Prep kit. Sequences were analyzed on an Illumina NextSeq 500 (Oneida) or HiSeq (IGB, A10) (Novogene, Hong Kong). Raw data are permanently stored on the NCBI Short Read Archive (accession numbers SRR9040995-8 and SRR10426055-76; Paraskevopoulou et al. 2019, 2020).

Using iterative mapping, mtgenomes were reconstructed with MITObim v.1.8 (Hahn et al. 2013) using default parameters and a mismatch value of 3–25. We used both available *B. calyciflorus* mtgenomes as bait for 2–10 independent runs with 1–9 iterations per run: Netherlands (GenBank accession MN417951.1/MN417952.1) and China (KX822781.1/KX822782.1). Consensus sequences for each independent run were constructed with ANGSD v.0.95 specifying the most common base call option. Resulting consensus sequences were aligned using ClustalW (Thompson et al. 2002), and final consensus sequences were called using a 75% base call threshold. Automatic annotation was performed with MITOS (Bernt et al. 2013). The complete mtgenomes varied in length from 27,413 to 28,162 bp for chromosome I and from 9961–9988 bp for chromosome II, in line with previously published mtgenomes of *B. calyciflorus*. All protein-coding genes, tRNAs and rRNAs were found (GenBank accession MZ706949, MZ706950 (Germany, IGB), MZ706951, MZ706952 (USA, Oneida)). With the same approach, the 12 protein-coding genes were identified for *B. fernandoi* (GenBank accession only for cds MZ768793–MZ768804). These sequences were aligned with other *Brachionus* species from GenBank using ClustalW. A maximum-likelihood phylogenetic tree was constructed using RaxML v.8 (Stamatakis 2014), performing 1000 bootstrap replicates with *Rotaria rotatoria* (Pallas, 1766) as an outgroup.

The phylogenetic analysis grouped both the German and the USA *B. calyciflorus* sensu stricto together with previously published *B. calyciflorus* specimens, suggesting that these mtgenomes derived from *B. calyciflorus* sensu stricto. *B. fernandoi* was placed with 100% bootstrap support as sister to the *B. calyciflorus* sensu stricto clade (Figure 1). This phylogenetic grouping, together with the average pairwise nucleotide divergence, illustrates that *B. calyciflorus* sensu stricto lineages across the world (i.e. Europe, China, and USA) are genetically much closer to each other (0.079 ± 0.033) than to their sister species *B. fernandoi* (0.254 ± 0.010). Our new mtgenomes constitute a resource for future studies on the *B. calyciflorus* species complex and support the monophyly of the globally distributed *B. calyciflorus* sensu stricto.

**Figure 1.**
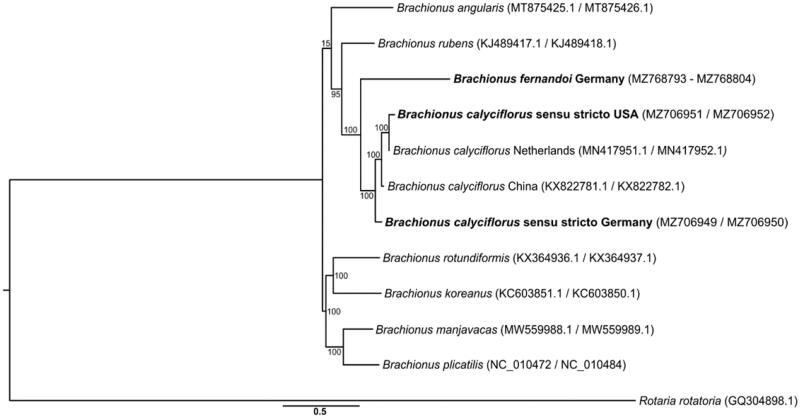
Maximum-likelihood tree based on full protein coding sequences of *Brachionus* rotifers. Adding our new genomes (bold) highlights the monophyly of both the *B. calyciflorus* species complex (albeit so far only represented by two of the four species) and the *B. calyciflorus* sensu stricto. The numbers on branches represent percentage bootstrap support.

## Data Availability

The genome sequence data that support the findings of this study are openly available on GenBank of NCBI at https://www.ncbi.nlm.nih.gov/ under the accession no MZ706949, MZ706950 (*B. calyciflorus* sensu stricto, Germany), MZ706951, MZ706952 (*B. calyciflorus* sensu stricto, USA), MZ768793–MZ768804 (*B. fernandoi*, Germany). The associated BioProject, SRA, and Bio-Sample numbers are PRJNA541384, PRJNA544636, SRR9040995–SRR9040998, SRR10426055–SRR10426076, and SAMN11584680–SAMN115846803, SAMN11845726–SAMN11845747, respectively.
